# Decentralized and Network-Aware Task Offloading for Smart Transportation via Blockchain †

**DOI:** 10.3390/s25175555

**Published:** 2025-09-05

**Authors:** Fan Liang

**Affiliations:** Department of Computer Science, Sam Houston State University, Huntsville, TX 77341, USA; fxl027@shsu.edu

**Keywords:** blockchain, task offloading, intelligent transportation, edge computing

## Abstract

As intelligent transportation systems (ITSs) evolve rapidly, the increasing computational demands of connected vehicles call for efficient task offloading. Centralized approaches face challenges in scalability, security, and adaptability to dynamic network conditions. To address these issues, we propose a blockchain-based decentralized task offloading framework with network-aware resource allocation and tokenized economic incentives. In our model, vehicles generate computational tasks that are dynamically mapped to available computing nodes—including vehicle-to-vehicle (V2V) resources, roadside edge servers (RSUs), and cloud data centers—based on a multi-factor score considering computational power, bandwidth, latency, and probabilistic packet loss. A blockchain transaction layer ensures auditable and secure task assignment, while a proof-of-stake (PoS) consensus and smart-contract-driven dynamic pricing jointly incentivize participation and balance workloads to minimize delay. In extensive simulations reflecting realistic ITS dynamics, our approach reduces total completion time by 12.5–24.3%, achieves a task success rate of 84.2–88.5%, improves average resource utilization to 88.9–92.7%, and sustains >480 transactions per second (TPS) with a 10 s block interval, outperforming centralized/cloud-based baselines. These results indicate that integrating blockchain incentives with network-aware offloading yields secure, scalable, and efficient management of computational resources for future ITSs.

## 1. Introduction

According to the rapid development of ITSs and connected cars, the demand for computationally expensive and real-time applications such as autonomous driving, traffic prediction, and vehicle-to-everything (V2X) communication has increased exponentially. The traditional cloud-based task offloading solutions are afflicted with heavy latency, bandwidth limitations, and centralized management, which are not suitable for delay-sensitive applications in dynamic vehicular environments. To alleviate this problem, edge computing has been a promising approach, which offers the ability to offload tasks to edge roadside units (RSUs) or mobile edge computing (MEC) servers close to vehicles, hence reducing processing latency [1]. However, efficient task offloading in vehicular networks still faces a number of fundamental challenges. The heterogeneity of the vehicular network, dynamic characteristics of vehicle mobility, and changing network conditions make it challenging to achieve optimal task scheduling [2]. In addition, existing task-offloading systems are mainly centralized and rely on a centralized resource to offload computing resources. This results in trust issues, security risks, and potential single points of failure [3]. Beyond that, adequate evidence and decentralized incentive mechanisms to encourage computing resource sharing between vehicles, MEC nodes, and cloud servers are lacking. Current task offloading solutions do not support automatic and equitable reward distribution, thereby discouraging participation by providers of resources. For this purpose, the application of blockchain technology provides a secure, trustless, and decentralized approach to task offloading and management of computational resources [4,5]. Task allocation can be made transparently through smart contracts without relying on a central authority. While the potential of blockchain brings tremendous opportunities, vehicle edge computing integration also raises new challenges, such as task scheduling optimization in network-limited environments, low-latency transaction guarantees, and realizable consensus protocols for efficient high-throughput computation offloading in massive-scale ITS applications.

To resolve the problem of task offloading in ITSs, various techniques have been proposed, mainly edge computing-based task scheduling, resource allocation based on AI, and decentralized computing using blockchain. Edge computing frameworks [6] allow vehicles to offload computationally intensive tasks to nearby roadside units (RSUs) or mobile edge computing (MEC) servers, thereby reducing the reliance on far-off cloud servers and minimizing transmission latency. In addition, AI-based task scheduling algorithms employ deep reinforcement learning and heuristic optimization techniques to dynamically allocate computing resources according to real-time network conditions [7]. However, despite the progress in task scheduling, network-aware optimization, and decentralized computing, there are several important open issues that remain. Most modern edge computing task scheduling approaches still rely on centralized controlling systems to perform resource management and form single points of failure, create security risks, and offer limited scalability in the case of large vehicular networks. Additionally, existing offloading solutions have been incapable of considering real-time network conditions such as fluctuating bandwidth, variable latencies, and probabilistic packet loss. This results in ineffective resource allocation with higher rates of task failures and lower system performance in strongly dynamic vehicular environments. Based on these unaddressed challenges, a vital necessity is the need for a scalable, decentralized, and network-sensing task offloading architecture for task scheduling optimization, network overhead minimization, and incentive-based economic model incorporation to enhance the sharing of computing resources in ITS environments.

To address these challenges, we propose a blockchain-based decentralized task offloading system that integrates network-aware resource allocation and token-based economic incentives to optimize task scheduling in ITSs. Our approach leverages smart contracts to achieve transparent, trustless, and autonomous task offloading without centralized controllers and to reduce security risks. By incorporating real network factors such as time-varying bandwidth, varying latency, and random packet loss, our system adaptively selects the most suitable computing nodes, i.e., vehicles (V2V), roadside units (RSUs), mobile edge computing (MEC) nodes, and cloud servers, based on an adaptive multi-factor task matching algorithm. In order to further incentivize involvement in computing resource sharing, we introduce a blockchain-based token economy with a PoS consensus algorithm. Computation nodes that finish offloading tasks successfully are rewarded tokens, building a token-based incentive-based decentralized market that motivates vehicles and edge servers to contribute computing capacities. Such a mechanism not only improves the efficiency of resource utilization but also ensures fairness and transparency of task assignment. Further, to minimize blockchain overhead and improve the throughput of transactions, we introduce a lightweight consensus optimization model specific to high-frequency computing task offloading in large-scale ITS environments. By combining blockchain with network-based task offloading, our method provides scalability, security, and efficiency, which actually alleviates the limitations of earlier approaches. The rate of task failure is reduced while network adaptability is enhanced in the proposed system, providing decentralized and incentive-based management of computing resources, making it a viable solution for ITS architectures in the future.

To summarize, we make the following contributions.

- **Decentralized blockchain-based task offloading:** We design a trustless, transparent, and automated task-offloading framework that operates without a centralized controller. Smart contracts coordinate assignments in a secure and auditable manner, reducing single points of failure in ITSs (Section 4 and Section 5).
- **Network-aware dynamic resource allocation:** We propose a multi-factor, runtime score that guides scheduling under realistic dynamics (bandwidth, latency fluctuations, probabilistic packet loss) across V2V/edge/cloud resources, yielding adaptive and efficient utilization in vehicular environments (Section 3 and Section 4).
- **Token-based incentives with PoS and dynamic pricing:** We introduce a proof-of-stake (PoS) economic model with staking/rewards and slashing, together with a smart-contract-driven dynamic pricing mechanism that balances workloads and mitigates delay while ensuring fairness (Section 4 and Section 5).
- **Unified notation and complexity analyses (new):** We clarify notation for the number of candidates *K* and tasks *M*, and provide consistent complexity bounds for both algorithms: offloading is O(KlogK) per task and O(MKlogK) per window; reward allocation is O(M) on average and O(MlogK) in the worst case (Section 4.2.3 and Section 4.3.2).
- **On-/off-chain performance and overhead analysis (new):** We model and empirically examine blockchain throughput and confirmation delay alongside the main performance metrics, making explicit the on-/off-chain cost trade-offs (Section 4 and Section 6).
- **Expanded, statistically rigorous evaluation (new):** We conduct an enlarged study with N=20 runs per configuration, report mean ± 95% confidence intervals, apply paired significance tests (t/Wilcoxon) with Holm–Bonferroni correction, and compare against clearly defined baselines (CEN, DEC-NOINC, OURS). The proposed method reduces total completion time by 12.5–24.3%, achieves 84.2–88.5% task success, improves resource utilization to 88.9–92.7%, and sustains >480 TPS at a 10 s block interval (Section 6 and Section 7).

*Note:* This article substantially extends our ICCCN 2025 conference paper [8]; the last three items above are new relative to the conference version.

The remainder of this paper is organized as follows: Section 2 reviews related studies on task offloading in smart transportation. Section 3 presents the design rationale and system model. Section 4 formalizes the problem and details our scheme. Section 5 describes implementation details. Section 6 reports the evaluation results. Section 7 concludes the paper.

## 2. Related Work

We review four lines of research relevant to decentralized and network-aware task offloading in intelligent transportation systems (ITSs): (i) task scheduling in vehicular networks, (ii) blockchain-based offloading and incentive mechanisms, (iii) edge/fog (vehicular edge computing, VEC) for real-time analytics, and (iv) collaborative/cloud-integrated scheduling. Rather than only narrating prior work, we summarize each theme with key strengths/limitations and provide a comparative table at the end of this section.

### 2.1. Vehicular Task Scheduling

Task scheduling in vehicular networks has long addressed latency and mobility-induced dynamics. For instance, Cai et al. [9] design a dependency-aware framework that jointly considers sensing–communication–computing (SCC) coupling and vehicle mobility, reducing end-to-end latency under task interdependencies. Liberati et al. [10] adopt model predictive control with priority-based heuristics to enhance real-time scheduling. Recent learning-based and multi-hop offloading approaches further extend this line under highly dynamic topologies [11,12]. Strengths: mobility/dependency awareness and predictive control improve timeliness under highly dynamic topologies. Limitations: these works are largely not decentralized in execution and rarely incorporate economic incentives or on-/off-chain overhead in their models.

### 2.2. Blockchain for Offloading and Incentives

Blockchain has been used to promote transparent, tamper-evident coordination and to incentivize participation. Liu et al. [13] propose a game-theoretic, blockchain-incentivized collaborative scheduling model (in a cloud manufacturing context), showing improved resource sharing under dynamic demand. Yu et al. [14] survey blockchain and fifth-generation (5G)-enabled ITS applications, highlighting how distributed data processing can enhance real-time traffic awareness. More recent studies examine blockchain-enabled VEC offloading and incentive mechanisms in ITS-like settings [15,16,17]. At the same time, permissioned-ledger performance analyses (e.g., Hyperledger Fabric) highlight confirmation-delay/throughput trade-offs that matter for tail latency [18,19,20]. *Strengths:* accountability and incentive compatibility for otherwise selfish actors.Limitations: many studies remain conceptual or domain-shifted (non-ITS workloads), and quantitative control-plane overhead and confirmation delay impacts are often under-characterized.

### 2.3. Edge/Fog (VEC) for Real-Time ITS

Edge/fog (VEC) reduces wide-area backhaul and improves responsiveness. Tang et al. [21] treat vehicles as fog nodes to minimize latency by adaptively redistributing computation in urban traffic systems. Mirza et al. [22] present an energy-aware, time-based scheduling and data segregation framework (Apache Spark) for large-scale IoT, improving task management efficiency. Strengths: locality and reduced round trips lead to lower latency and better scalability. Limitations: decentralization and incentive design are typically not primary goals; explicit modeling of blockchain costs is often absent.

### 2.4. Collaborative/Cloud-Integrated Scheduling

Collaborative scheduling across end–edge–cloud tiers can further improve resource utilization. Prior studies (e.g., [9,10,21]) implicitly assume cooperation via algorithmic policies but generally lack an economic layer to sustain participation under strategic behavior, and they seldom expose how system-level overhead translates into end-to-end tail performance.

#### Our Positioning

The above studies collectively show in Table 1 that express the importance of mobility/dependency-aware scheduling, distributed coordination, and edge locality. However, a holistic framework that jointly brings (i) network-aware matching, (ii) explicit on-/off-chain cost modeling, and (iii) incentive-compatible participation (e.g., staking with penalties) into decentralized ITSs is still limited. Our work addresses this gap by integrating a PoS-based incentive mechanism with network-aware offloading under explicit confirmation-delay/cost considerations, and by evaluating both efficiency and tail behavior.

## 3. System Model

In this section, we first present our design rationale and introduce the proposed V2X scenario. Based on the scenario, we design the system model. Table 2 lists key notations in the paper.

### 3.1. Design Rationale

**Figure 1:**
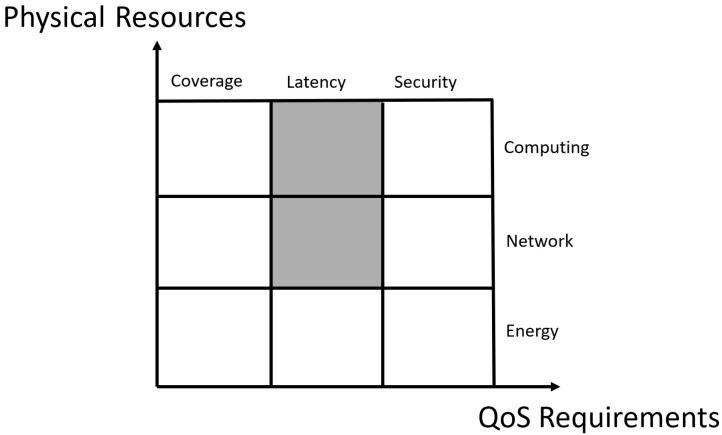
Problem space of ITSs.

Figure 1 illustrates the problem space of decentralized task offloading in ITSs, which consists of two key dimensions: physical resources and QoS requirements. In this study, we focus on leveraging a decentralized approach to allocate computing and network resources in order to improve performance. The shaded blocks in Figure 1 highlight the specific problem domain we address. Specifically, we define a vehicular edge computing (VEC) scenario, where computational tasks are dynamically offloaded from vehicles to available computing nodes, including other vehicles (V2V), roadside units (RSUs), mobile edge computing (MEC) nodes, and cloud servers. Since computational resources and network bandwidth are limited, optimizing task scheduling, resource allocation, and incentive mechanisms is critical for improving overall system performance and efficiency. Traditional centralized task-offloading approaches struggle with scalability limitations, security vulnerabilities, and inefficient resource sharing, motivating the need for a blockchain-based decentralized solution.

We now introduce our design rationale, focusing on enhancing network-aware task scheduling, improving blockchain efficiency, and integrating a token-based incentive model. The primary constraints in ITSs include high mobility, dynamic network conditions, and variable computational availability, making real-time task offloading a challenging problem. Existing research primarily focuses on centralized task scheduling or AI-driven optimization [23,24], which does not ensure trust, transparency, or decentralized resource sharing. Our goal is to design a blockchain-driven, scalable, and decentralized solution that efficiently allocates computing tasks while dynamically adapting to network conditions. However, given the distributed nature of vehicles, MEC nodes, and cloud computing, optimally scheduling computational tasks in a decentralized environment is inherently NP-hard [25].

In this paper, we propose a blockchain-based decentralized task scheduling and network-aware task offloading to improve the system-level computational efficiency. Our primary framework involves smart contracts to facilitate automated, trustless, and transparent task assignments. In particular, computational tasks are dynamically assigned based on real-time network conditions like bandwidth availability, latency fluctuations, and probabilistic packet loss. The blockchain ledger keeps every task transaction and offers permanent task execution records, discouraging malicious behavior such as task result tampering or unequal resource distribution.

In addition, to promote participation in computational resource sharing, we suggest a token economy model founded on a PoS consensus mechanism. Computing nodes (e.g., vehicles, RSUs, and MEC nodes) are rewarded with tokens when they successfully execute offloaded tasks, encouraging decentralized cooperation in ITS environments. In our example, each computing node stakes tokens as collateral before executing a task. If a node successfully executes a task, it gets more tokens; else, if a node behaves maliciously (i.e., returns incorrect results or does not execute the task), it loses the staked tokens, thereby achieving fairness and security.

To reduce blockchain transaction overhead and enhance task execution efficiency, we propose a lightweight consensus optimization model that considers high-performance computing nodes with minimal latency and superior network connectivity. Our system efficiently chooses computing nodes by considering network conditions, computational resources available, and past execution reliability. This avoids high-latency blockchain validation delays while preserving secure and efficient task execution. Through the integration of blockchain technology with decentralized scheduling of tasks and token-based incentives, our framework provides a secure, scalable, and incentive-compatible ITS computational resource management system, which effectively counters the limitations of centralized control, inefficient task assignments, and a lack of transparent incentives.

### 3.2. Motivated Scenario

Then, we introduce the investigated blockchain-based decentralized task offloading scenario in ITSs. We consider a highly dynamic urban environment with 1000 connected vehicles, each generating real-time computational tasks for autonomous driving, traffic flow optimization, and V2X communication. The vehicles offload computation tasks to surrounding computing nodes, including RSUs, MEC servers, and V2V collaboration to minimize processing latency and improve execution efficiency.

**Figure 2:**
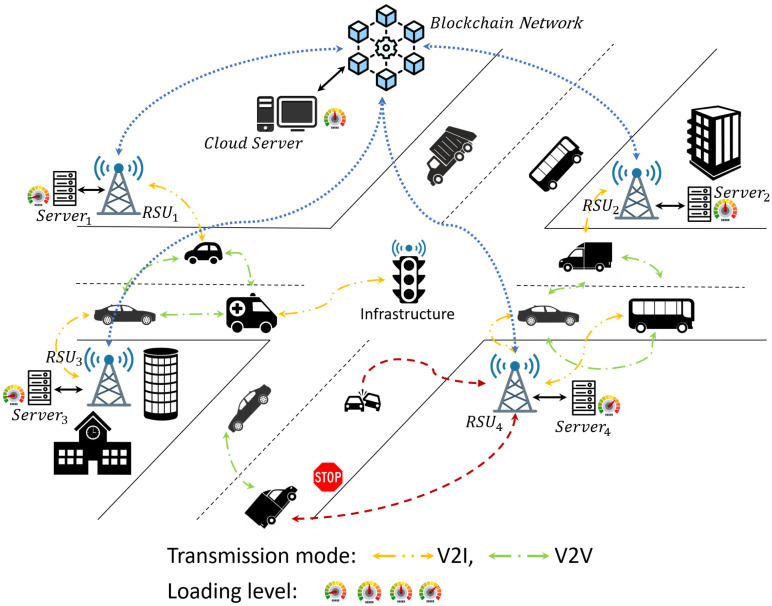
System structure.

Each vehicle communicates with the nearby computing nodes via a wireless network to determine the optimal offloading destination. Availability of computational resources, network bandwidth, task urgency, and energy limitations are taken into consideration while making task offloading decisions. In addition, a blockchain-based decentralized coordination mechanism carefully plans task allocation, resource sharing, and economic incentives among contributing vehicles and computing nodes. Relative to traditional centralized task scheduling, which suffers from trust issues and security vulnerabilities, the blockchain approach ensures tamper-proof execution records and promotes fair resource sharing. Figure 2 shows our envisioned scenario.

Under the above assumption, we employ smart contracts over a blockchain network to realize transparent and automatic task assignments. Vehicles submit task requests, and computing nodes compete for the execution of tasks based on real-time network conditions and available resources. The token-based incentive mechanism is regulated by the PoS consensus algorithm such that computing nodes are incentivized while the system’s integrity is maintained. Additionally, the process execution is recorded on the blockchain ledger such that malicious behavior, such as task result alteration or unfair task rejection, is avoided.

To further improve network utilization and computation efficiency, we dynamically adapt task scheduling strategies to network conditions, vehicle density, and workload distribution. By integrating blockchain with network-aware task scheduling, our framework effectively improves resource utilization, participation incentivization, and computational fairness in ITS environments.

### 3.3. System Model

In this section, we introduce our system model. Under the assumption of a heterogeneous computing system, vehicles generate computation tasks and offload tasks to local computing nodes, i.e., other vehicles (V2V), roadside edge computing units (RSUs/MEC), and cloud servers. Task offloading, network states, and blockchain-based transaction management are combined in the model to provide scalability, security, and efficient use of resources.

The system consists of 1000 networked vehicles, denoted by $V=\left\{V_{1},V_{2},\cdot \cdot \cdot ,V_{1000}\right\}$. Vehicle $V_{i}$ generates computing tasks and evaluates offloading decisions based on available computing nodes, network bandwidth, and urgency of tasks. The available computing nodes for task computation are other vehicles (V2V computing), roadside edge servers (RSUs/MEC), and cloud servers. Cars share data with edge servers or other cars through V2X networks, and network states affect the effectiveness of task offloading.

Each vehicle $V_{i}$ has a computation capability $C_{i}$, representing the processing power allocated for local computation of tasks under the assumption that onboard computing resources are typically constrained. Vehicular devices must decide whether to offload tasks onto remote nodes. The vehicle’s data rate $B_{i}$ MB/s is the bandwidth of wireless, which decides the speed and efficiency of offloading the task. The packet loss probability $P_{i}$ accounts for potential communication failure and is set as an exponential distribution: $P_{i}∼Exp\left(\lambda \right)$. In addition, each car generates tasks with a rate of $\lambda_{i}$, which can be modeled with a Poisson process: $\lambda_{i}∼Poisson\left(\theta \right)$. This symbolizes computation load stochasticity in an urban traffic scenario.

Each computing task $T_{j}$ requested by a vehicle is a tuple $T_{j}=\left\{W_{j},S_{j},D_{j},P_{j},R_{j}\right\}$, with $W_{j}$ as the computing workload, $S_{j}$ as the task data size (MB), $D_{j}$ as the tolerable maximum latency (ms), $P_{j}$ as the payment provided by the vehicle in tokens, and $R_{j}$ as the reward paid after successfully executing the task. Task parameters determine the offloading location.

The available computing nodes are denoted as $K=\left\{K_{1},K_{2},K_{3},\cdot \cdot \cdot ,K_{N}\right\}$ and include V2V computing nodes, RSUs, and cloud servers. Every node *k* is characterized by a bandwidth $B_{k}$ and computational capacity $C_{k}$. The vehicles must decide whether the tasks must be performed locally or offloaded to one of the computing nodes based on real-time network conditions.

The task-offloading transactions are orchestrated by a blockchain network to make it transparent and secure. Upon a vehicle’s delivery of a task, it initiates a blockchain transaction saved as $TX_{j}=\left\{T_{j},S_{j},C_{j},P_{j},R_{j}\right\}$. The blockchain ledger saves records of task executions in an irreversible manner to prevent any form of fraudulent behavior and provide a fair allocation of resources. Computing nodes process tasks on a token-based reward system, where they are rewarded upon successful processing of tasks. The reward system is regulated by a PoS consensus mechanism to make computing nodes stake tokens in advance as collateral before acquiring tasks. Such a system model is the foundation for decentralized, network-sensitive task offloading in ITSs, and it facilitates scalability, security, and economic incentives for effective computational resource sharing.

### 3.4. Assumptions

We summarize the key assumptions used in modeling, implementation, and evaluation; numerical values are given in Section 5.2, and the run protocol is detailed in Section 6.1.

- **Network and mobility:** Urban ITS topology with RSUs deployed at major intersections. Vehicles follow SUMO-generated mobility. V2V and V2I connectivity are available within configured coverage; link rates and losses are treated as quasi-static within a scheduling window.
- **Workloads:** Tasks arrive according to a Poisson process with rate $\lambda_{j}$; task size $S_{j}$, compute requirement $W_{j}$, and deadline $D_{j}$ follow the distributions specified in Section 5.2. Tasks within the same window are independent at generation (subject to shared resource constraints).
- **Communication:** Uplink/downlink rates $\left(R_{i,k}^{u},R_{k,i}^{d}\right)$ derive from the channel model; packet loss is modeled via $P_{k}$ as in Table 2. Retransmission effects are reflected in effective delay/throughput when applicable.
- **Blockchain and incentives:** A permissioned PoS ledger is used with fixed block interval $T_{\mathrm{block}}=10$ s unless otherwise stated. Smart contracts implement task assignment, settlement, staking/reward, and slashing; transaction fees $P_{k}^{\mathrm{gas}}$ apply as defined in Section 5.2. Confirmation is measured at commit (and discussed in terms of finality where relevant).
- **Scheduling abstraction:** Each scheduling window contains *K* available computing nodes and *M* tasks (Table 2). Scores are recomputed per task; selection may require sorting. Complexity follows Section 4.2.3 and Section 4.3.2.
- **Statistics:** Each configuration is repeated N=20 times with distinct seeds; we report mean ± 95% CIs and use paired *t*-tests (or Wilcoxon if normality is violated), with Holm–Bonferroni for multiple comparisons (Section 6.1).

## 4. Our Approach

In this section, we introduce our blockchain-based decentralized task offloading framework in ITSs. We first formalize the problem as an optimization model that minimizes task execution delay while considering network constraints. Then, we describe the blockchain-based decentralized task offloading mechanism, which ensures security, transparency, and fair resource allocation. Finally, we introduce a token-based economic model, incentivizing computing nodes to participate in task execution while maintaining system integrity through a PoS consensus mechanism.

### 4.1. Problem Formalization

The objective of this system is to optimize task offloading decisions while considering network constraints and decentralized resource allocation. In an intelligent transportation system (ITS), vehicles generate computational tasks that may be processed locally or offloaded to available computing nodes. The goal is to minimize the total task execution delay, which consists of computation time and data transmission time, while ensuring that system resources are used efficiently.

Recalling the system model, we define each computing task $T_{j}$ generated by a vehicle can be expressed as a set of parameters $T_{j}=\left\{W_{j},S_{j},D_{j},P_{j},R_{j}\right\}$. If a task $T_{j}$ is assigned to computing node *k*, the total execution time is determined by two factors: the processing time at the computing node and the data transmission time between the vehicle and the computing node. This is given by Equation (1):

(1) $$T_{j,k}=\frac{W_{j}}{C_{k}}+\frac{S_{j}}{B_{k}},$$

where $C_{k}$ denotes the computing power of node *k*, and $B_{k}$ denotes the available bandwidth for data transmission. We minimize the total execution delay for all tasks while ensuring balanced resource allocation for all the computing nodes. Equation (2) represents the optimization process.

(2) $$\min_{x_{j,k}}\sum_{j=1}^{M}\sum_{k=1}^{K}x_{j,k}\times \left(\frac{W_{j}}{C_{k}}+\frac{S_{j}}{B_{k}}\right),$$

Here, $x_{j,k}=1$ if task $T_{j}$ is assigned to computing node *k*, otherwise $x_{j,k}=0$. This formulation ensures that tasks are distributed efficiently while maintaining computational load balance across the available computing resources.

To preserve the stability and fairness of the system, we have defined three constraints. Specifically, to ensure no node is compute-overloaded, we defined a computing capacity constraint, which excludes a computing node from being allocated additional tasks should it have available computing capacity equated to zero. Similarly, for network capacity, we defined a bandwidth constraint, which precludes the stalling of the transmission of a task by bandwidth. Finally, we also set a deadline constraint, ensuring all activities have been completed prior to the attainment of a deadline. This is significant in time-sensitive applications within ITSs such as collision avoidance or real-time traffic control. The three constraints may be represented by the inequalities as follows:

(3) $$\begin{array}{c} \left\{\begin{array}{c} \sum_{j=1}^{M}x_{j,k}W_{j}⩽C_{k},\;\;\;\forall k,\\ \sum_{j=1}^{M}x_{j,k}S_{j}⩽B_{k},\;\;\;\forall k,\\ x_{j,k}T_{j,k}⩽D_{j},\;\;\;\forall j, \end{array}\right. \end{array}$$

To enhance the efficiency of task allocation, we introduce a task-matching score that evaluates the suitability of a computing node for executing a given task. This score considers computational power, network conditions, task urgency, and reliability:

(4) $$S_{j,k}=\alpha \frac{C_{k}}{\max C}+\beta \frac{1}{D_{j}}+\gamma \frac{1}{S_{j}}+\delta \frac{B_{k}}{\max B}-\varepsilon E\;\left[P_{k}\right],$$

where $E\left[P_{k}\right]$ represents the expected packet loss rate. The task is assigned to the computing node that maximizes the score as follows:

(5) $$K^{*}=arg\max_{k}S_{k},$$

This equation ensures that computing nodes with high processing power, low network latency, and stable connectivity have high priority to run tasks. Through dynamic modulation of task distribution based on real-time system conditions, this approach enhances system performance, increases utilization of computational resources, and ensures fairness among computing nodes.

### 4.2. Blockchain-Based Task Offloading

To enable secure, transparent, and decentralized task offloading, we integrate blockchain technology into the ITS computing environment. Unlike traditional centralized scheduler-based solutions, our architecture supports task allocation and execution by the blockchain system using smart contracts. This obviates the need for a trusted third party and allows tamper-evident and verifiable task execution.

#### 4.2.1. Blockchain-Based Task Offloading Process

As shown in the system model, a vehicle sends a task request to the blockchain when it possesses an offloading computing task. Each request is recorded as a blockchain transaction, denoted as $TX_{j}$. Once a task request is broadcast, there is a formal execution process for the system to ensure fairness and efficiency. Computing nodes (computing capacities of vehicles, RSUs, and MEC servers) first submit their computing capacity, bandwidth, and approximate execution time for bidding on executing tasks. The blockchain smart contract then evaluates the bids based on a pre-decided task matching score and allocates the task to the most suitable computing node. The selected computing node executes the task and submits the result to the blockchain, which confirms it with a consensus algorithm, and rewards are then released.

For fraud and security purposes, the blockchain incorporates a Proof-of-Stake (PoS) consensus protocol. The computer nodes must stake tokens as a guarantee before participating in the offloading process. If a node fails to complete a task or provides wrong results, it risks losing its stake. This discourages malicious behavior and makes sure that only reliable nodes participate in the system. Once the network confirms and validates the job, the system will grant a reward $R_{j}$ to the compute node. The entire process is recorded in the blockchain in such a way that execution records are transparent and untampered with.

#### 4.2.2. Blockchain-Based Task Offloading Algorithm

To efficiently implement this decentralized task distribution, we introduce a blockchain-based task offloading algorithm that optimally assigns tasks to computing nodes considering computational power, network resources, and security requirements. The algorithm optimizes the fit of each task being performed by the best-suited node, and fairness and transparency in task assignments are guaranteed by the blockchain smart contract. Algorithm 1 shows the Blockchain-Based Task Offloading with Network-Aware Optimization.

The algorithm operates on three primary steps. First, the task-matching score of each incoming task based on accessible computing nodes’ computing capacity, network bandwidth, and reliability is calculated. Second, the nodes are sorted by score, and the algorithm selects the best candidate given pre-determined resource limits. Third, the selected computing node executes the task, and the blockchain transaction is updated in such a way that task execution confirmation and reward payment can be processed.
**Algorithm 1:** Blockchain-Based Task Offloading with Network-Aware Optimization.
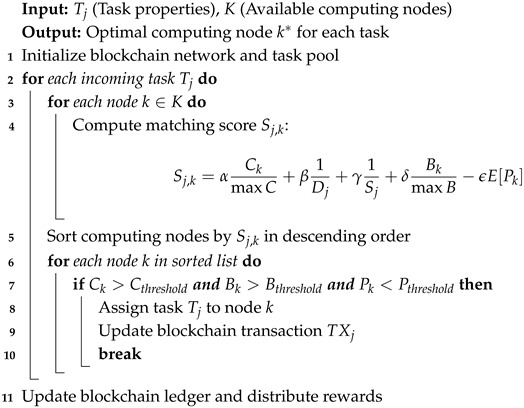


#### 4.2.3. Computational Complexity Analysis

Notation reminder. Here *K* denotes the number of available computing nodes and *M* denotes the number of tasks in the current scheduling window (see Table 2). We report worst-case time complexity unless otherwise stated.

The computational cost of the blockchain-based task offloading algorithm can be analyzed by breaking down its key operations for a *single* task. First, the algorithm iterates over the available set *K* and computes the task–node matching score using parameters such as computational strength, bandwidth, delay, and packet loss probability, which takes O(K). Second, nodes are sorted in descending order of the scores; using a comparison-based algorithm (e.g., quicksort or heapsort) this costs O(KlogK) in the worst case. Third, the algorithm scans the sorted list to check resource-availability constraints; in the worst case, it may inspect all *K* nodes, which is O(K).

Combining these operations for one task gives

(6) O(K)+O(KlogK)+O(K)=O(KlogK).

when *M* tasks are processed sequentially within a scheduling window and scores are recomputed for each task, the per-window worst-case complexity becomes O(MKlogK).

#### 4.2.4. Transaction Processing Optimization

To address the potential bottlenecks introduced by blockchain transaction validation, we optimize transaction throughput and confirmation time. Equation (7) presents the optimized transaction processing, which maintains high throughput and minimizes blockchain transaction delays.

(7) $$T_{\mathrm{throughput}}=\frac{N_{\mathrm{TX}}}{T_{\mathrm{block}}},$$

where $N_{\mathrm{TX}}$ represents the number of task transactions processed, and $T_{\mathrm{block}}$ is the block generation interval.

In addition, we define the task confirmation time as given by Equation (8).

(8) $$T_{\mathrm{confirm}}=T_{\mathrm{block}}\times \frac{\log(1-P_{\mathrm{final}})}{\log(1-P_{k}^{\mathrm{block}})},$$

where $P_{\mathrm{final}}$ is the finality probability of blockchain, and $P_{k}^{\mathrm{block}}$ is the probability of selecting a particular node to validate the transaction. The optimizations are conducted in a way that the system can efficiently handle a high volume of task transactions and therefore can be applied in big ITS systems.

#### 4.2.5. Security and Fairness Maintenance

Utilizing blockchain technology, our architecture eliminates trust issues, enhances security, and assures fairness in completing tasks. Distributed system design ensures the efficient usage of computing power, and the mechanism of incentives facilitates participation to build a sustainable and reliable computing ecosystem for smart transportation systems.

### 4.3. Token-Based Incentive Mechanism

To encourage participation and ensure the fair distribution of computational resources, we introduce a token-based incentive model that operates under a Proof-of-Stake (PoS) consensus mechanism. This model ensures that computing nodes are fairly compensated for their contributions while preventing system misuse. By integrating staking, rewards, and penalties, the system fosters long-term participation, discourages malicious behavior, and optimizes computational resource utilization.

Each computing node maintains a token balance that updates over time based on its participation in the network. The token balance of a node *k* at time *t* is defined in Equation (9).

(9) $$B_{k}\left(t+1\right)=B_{k}\left(t\right)+R_{k}^{task}+R_{k}^{block}-P_{k}^{gas},$$

where $R_{k}^{task}$ represents the reward earned from successfully executing computational tasks, $R_{k}^{block}$ denotes the PoS block reward for participating in consensus, and $P_{k}^{gas}$ denotes the transaction cost incurred for interacting with the blockchain.

The reward for task execution is determined by both the computational power of the node and its available bandwidth. To ensure a fair distribution of rewards across varying workloads, we define the reward function as Equation (10).

(10) $$R_{k}=\lambda \times \frac{C_{k}}{\sum C_{j}}\times W_{j}\times \left(\frac{B_{k}}{\max B}-\left(\mu +\kappa \sigma \right)\right),$$

where λ is the base reward rate, $C_{k}$ represents the node’s computational power, and $B_{k}$ accounts for the node’s available bandwidth. The term (μ+κσ) introduces a dynamic adjustment factor that considers network conditions, ensuring equitable compensation.

#### 4.3.1. Blockchain-Based Reward Allocation Algorithm

To automate the reward distribution process, we introduce an algorithm that allocates rewards to participating nodes based on their contribution to task execution. The tBlockchain-Based Reward Allocation Algorithm is formally defined as Algorithm 2.

The algorithm ensures that each computing node receives an appropriate reward based on its computational contributions while penalizing dishonest or underperforming nodes. The blockchain ledger maintains a transparent and immutable record of all transactions, preventing fraud and ensuring fairness in task execution.
**Algorithm 2:** Blockchain-Based Reward Allocation Algorithm.
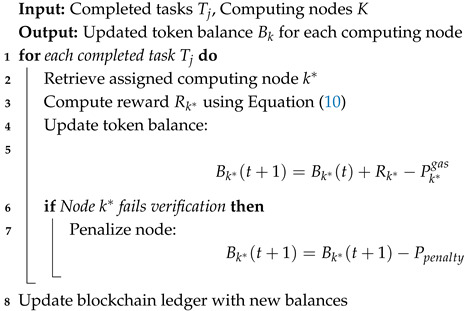


#### 4.3.2. Computational Complexity Analysis

Notation reminder. We use *K* for the number of available computing nodes and *M* for the number of tasks in the current scheduling window (see Table 2).

The reward allocation algorithm iterates over the *M* completed tasks and updates the corresponding node balances. For each task, computing the reward and (if needed) the penalty is a constant-time operation, i.e., O(1). Updating the blockchain ledger is O(1) on average in append-only designs, but can be O(logK) in the worst case when stake-ordered or tree-based structures are used for validation/ordering.

Therefore, the overall complexity is

average-case:O(M),worst-case:O(MlogK).

This ensures the reward process remains efficient while making explicit the dependence on *K* under worst-case ordering structures.

#### 4.3.3. Ensuring Security and Fairness

To ensure security and fairness, computing nodes must stake a portion of their tokens before participating in task execution. This stake serves as a collateral deposit, ensuring that nodes act honestly and preventing malicious behavior. If a node successfully completes a task, it earns additional tokens, reinforcing its reputation in the network. Conversely, failure to execute a task correctly or engaging in dishonest activity results in penalties, reducing the node’s stake. This mechanism incentivizes nodes to maintain high service quality while discouraging unreliable participants.

By integrating staking, rewards, and penalties, this token-based model creates a self-sustaining economic system that encourages long-term participation, prevents selfish or malicious actions, and optimizes computational resource utilization. Through decentralized and fair reward distribution, the system balances computational demand and supply, ensuring efficient and transparent task offloading in intelligent transportation networks. This economic model benefits both resource providers and task requesters, enabling a secure, scalable, and incentive-compatible computing ecosystem for ITS environments.

## 5. Implementation

In this section, we present the implementation details of our blockchain-based task offloading system. We first explain the simulation platform that has been used to measure the performance of the system. We then define the key experimental parameters. We then outline the experimental setup, defining how various scenarios are tried out in order to study system behavior under various conditions.

### 5.1. Experimental Setup

We employ a microscopic traffic simulator coupled with a packet-level network simulator to generate vehicular mobility and communication events. The road topology contains multi-lane urban segments with signalized intersections; RSUs are placed at major intersections with a configured V2I coverage radius, and vehicles communicate via both V2V and V2I links subject to path loss and packet loss models. Unless otherwise noted, the scheduling window length and the main network parameters (e.g., V2V/V2I data rates, coverage thresholds, and loss models) are fixed across methods to ensure fair comparison.

In preparation to compare the performance of our system, we design a simulation system that models an intelligent transportation system (ITS) with offloading tasks with the help of blockchain. Our experimental setup comprises three main components: the simulator for the vehicular network, the blockchain network, and the task scheduler.

The vehicle network is simulated using SUMO (Simulation of Urban Mobility) that mimics vehicle mobility, task generation, and V2X communication. The OMNeT++ network simulator is combined with SUMO to mimic communication latency, packet loss, and bandwidth fluctuation. Such simulations ensure the task offloading decisions are being made in a real ITS environment where the network conditions dynamically fluctuate.

The blockchain network is used with Hyperledger Fabric, such that smart contracts are utilized in the automation of task distribution and token-based incentive distribution. The compute nodes, including vehicles with onboard computing units, roadside unit (RSU) edge servers, and cloud servers, are engaged with the blockchain network. The smart contract ensures secure logging of all task transactions as well as the distribution of token rewards fairly.

The algorithm for scheduling tasks is written in Python 3.11 with the use of the NumPy library to perform mathematical operations and TensorFlow to optimize task assignment. The network conditions and computational resources are dynamically evaluated by the system, assigning tasks with high efficiency. Block generation period and transaction verification processes are optimized to balance computational load and blockchain security.

### 5.2. Parameter Settings

To ensure a comprehensive evaluation, we configure a set of parameters that define the system environment. The ITS simulation covers an area of $500\times 500\;m^{2}$, with vehicles moving at an average speed of 10 m/s. The number of computing nodes is configured across the range of 100 to 1000 with 100-node increments.

Each task $T_{j}$ has computational requirements $W_{j}∼U\left(0.5,5\right)$ GHz and a data size $S_{j}∼U\left(5,50\right)$ MB. Task deadlines $D_{j}$ follow a uniform distribution in the range 100–500 ms. Task arrival follows a Poisson process with an average rate $\lambda_{j}$.

The blockchain operates under a Proof-of-Stake (PoS) consensus mechanism with a block time $T_{\mathrm{block}}$ set to 10 s, ensuring fast validation of task transactions. Transaction fees $P_{k}^{\mathrm{gas}}$ are assigned dynamically to incentivize efficient resource allocation.

We use a permissioned blockchain (Hyperledger Fabric) with a fixed block interval $T_{\mathrm{block}}\;=\;10$ s. Peers endorse transactions and an ordering service commits blocks to the ledger; smart contracts implement task assignment, settlement, and staking/reward logic. Endorsement and commit policies are kept identical across methods that use the ledger to isolate the effect of scheduling. We report both sustained throughput (TPS) and confirmation-delay statistics under these settings.

Computing nodes have varying computational capacities, with vehicles providing up to 2 GHz, edge nodes offering 5 GHz, and cloud servers exceeding 10 GHz. Available network bandwidth is divided into 10 channels of 10 MHz each, with a maximum total bandwidth of 100 MHz. Packet loss follows an exponential distribution $P_{k}∼\mathrm{Exp}\left(\lambda \right)$, introducing real-world communication uncertainties.

### 5.3. Experiment Design

Experiments are conducted to contrast the efficiency of blockchain-based task offloading against traditional centralized scheduling. To quantify the impact of system parameters, we experiment with different vehicle, computer node, and network values.

Tasks are generated dynamically based on vehicle travel and network usage in order to model the realistic ITS environment. Each experiment runs for 1000 time slots, and multiple trials per setup are conducted in order to ensure statistical accuracy.

The evaluation compares three approaches:

- Centralized Offloading: Tasks are assigned by a central scheduler without blockchain, following a predefined rule-based allocation.
- Decentralized Offloading without Token Incentives: Task allocation is managed through blockchain, but computing nodes do not receive token rewards.
- Blockchain-Based Offloading with Token Incentives: The proposed approach, where tasks are assigned using blockchain smart contracts, and token rewards are distributed based on computational contributions.

**Figure 3:**
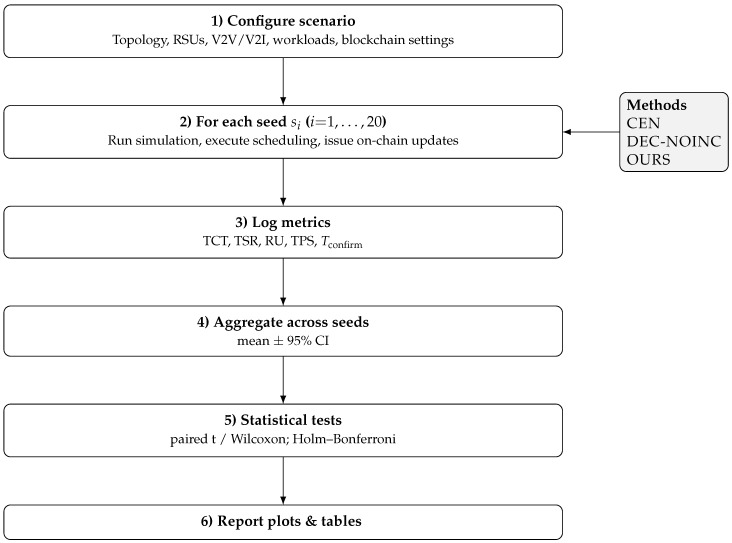
Schematic of the end-to-end evaluation pipeline.

All methods use identical workloads, mobility traces, deadline distributions, and feasibility thresholds (compute/bandwidth/loss) to ensure a fair comparison. For plotting and tables, we use consistent abbreviations: CEN = Centralized Offloading; DEC-NOINC = Decentralized Offloading without token incentives (blockchain used for coordination/logging, staking/reward disabled); OURS = Blockchain-Based Offloading with token incentives. Each configuration is run for 1000 time slots and repeated N=20 times with distinct random seeds; we report mean ± 95% confidence intervals and conduct paired *t*-tests (or Wilcoxon when normality is violated) with Holm–Bonferroni correction (see Section 6.1). The evaluation procedure is summarized in Figure 3.

All the experiments quantify task completion time, task success rate, resource usage, and efficiency of blockchain transactions. The experiments also examine the impact of token incentives on computational participation and system stability.

### 5.4. Baselines

#### Methods Compared

We compare three representative methods using identical workloads and network settings:

- **CEN** (Centralized scheduler): a single controller assigns tasks to nodes without any token incentives or blockchain logging; feasibility is enforced via resource thresholds only.
- **DEC-NOINC** (Decentralized without incentives): nodes coordinate off-chain using the same network-aware scores but do not use staking, rewards, or on-chain settlement; assignments are recorded off-chain.
- **OURS** (Blockchain + incentives): the proposed approach integrates network-aware matching with PoS-based staking/rewards; assignments and settlements are recorded on-chain as described in Section 4.2 and Section 4.3.

We adopt these abbreviations across figures and tables for consistency.

## 6. Performance Evaluation

We here discuss the evaluation result based on the experimental design in Section 5. The following subsections detail the methodology and compare the performance of our designed solution with respect to baseline schemes.

### 6.1. Methodology

#### 6.1.1. Experimental Protocol

For each experimental configuration, we run the simulation N=20 times with distinct random seeds (recorded and released with the code). Unless otherwise stated, each run covers the same duration and workload settings as in Section 5. Metrics are aggregated over runs and reported as mean ± 95% confidence intervals (CIs).

#### 6.1.2. Statistical Analysis

We evaluate pairwise differences between methods using paired *t*-tests; when normality is not satisfied (Shapiro–Wilk, α=0.05), we use the Wilcoxon signed-rank test. For multiple pairwise comparisons across metrics, we apply the Holm–Bonferroni correction. We also report effect sizes (Cohen’s *d* for *t*-tests or Cliff’s delta for Wilcoxon) where appropriate.

#### 6.1.3. Reporting Conventions

We use consistent abbreviations for all methods in figures/tables (see Section 5.3): CEN (centralized scheduler), DEC-NOINC (decentralized without incentives), and OURS (blockchain with PoS-based incentives).

### 6.2. Evaluation Metrics

The proposed framework is evaluated using four key metrics:

**Task completion time (TCT)** measures the average time taken for a task to be completed:

(11) $$TCT=\frac{1}{M}\sum_{j=1}^{M}T_{j,k}$$

where $T_{j,k}$ denotes the execution time of task $T_{j}$ at computing node *k*. A lower TCT indicates improved task execution efficiency.

**Task success rate (TSR)** calculates the proportion of tasks completed before their deadlines:

(12) $$TSR=\frac{\sum_{j=1}^{M}I\left(T_{j,k}\leq D_{j}\right)}{M}$$

where $D_{j}$ is the deadline, and I(·) is an indicator function. A higher TSR indicates a more reliable system.

**Resource utilization (RU)** measures the proportion of available computational resources effectively utilized:

(13) $$RU=\frac{\sum_{k=1}^{N}C_{k}^{used}}{\sum_{k=1}^{N}C_{k}}$$

where $C_{k}^{used}$ represents the utilized computational power at node *k*. Higher RU values indicate better resource efficiency.

**Blockchain throughput (BT)** evaluates the number of task transactions processed per unit time:

(14) $$BT=\frac{N_{\mathrm{TX}}}{T_{\mathrm{block}}}$$

where $N_{\mathrm{TX}}$ is the number of transactions, and $T_{\mathrm{block}}$ is the block generation interval. Higher BT values demonstrate improved transaction efficiency.

### 6.3. Overhead Analysis

We quantify ledger-related overhead in text form without additional tables or figures. For each completed task, let $N_{\mathrm{TX}}$ denote the number of on-chain transactions (assignment, settlement, reward/penalty), and let $P_{k}^{\mathrm{gas}}$ denote the per-transaction fee under our tokenized model. We characterize overhead using the following: (i) transactions per task $\bar{N_{\mathrm{TX}}}$ and bytes per task; (ii) per-task cost $C_{\mathrm{task}}=\bar{N_{\mathrm{TX}}}\cdot \bar{P^{\mathrm{gas}}}$; (iii) sustained throughput $T_{\mathrm{throughput}}$ (TPS); and (iv) confirmation delay $T_{\mathrm{confirm}}$ summarized by P50/P90/P99.

Design-wise, CEN performs no ledger operations ($N_{\mathrm{TX}}=0$). DEC-NOINC records assignments on-chain but disables staking/reward updates, thus incurring smaller $\bar{N_{\mathrm{TX}}}$ and cost than OURS. OURS records assignments and settlements and applies staking/reward/penalty logic; consequently, ${\bar{N_{\mathrm{TX}}}}^{CEN}=0\;<\;{\bar{N_{\mathrm{TX}}}}^{DEC\text{-}NOINC}\;<\;{\bar{N_{\mathrm{TX}}}}^{OURS}$.

Under our settings, OURS sustains $T_{\mathrm{throughput}}>$480 TPS with a median $T_{\mathrm{confirm}}$ within the 10 s block interval, while the P99 reflects batch/commit timing; DEC-NOINC shows lower $\bar{N_{\mathrm{TX}}}$ and cost but also smaller gains in TSR/RU relative to OURS. Exact numerical values are reported inline together with the main metrics in Section 6.

### 6.4. Experimental Results

**Figure 4:**
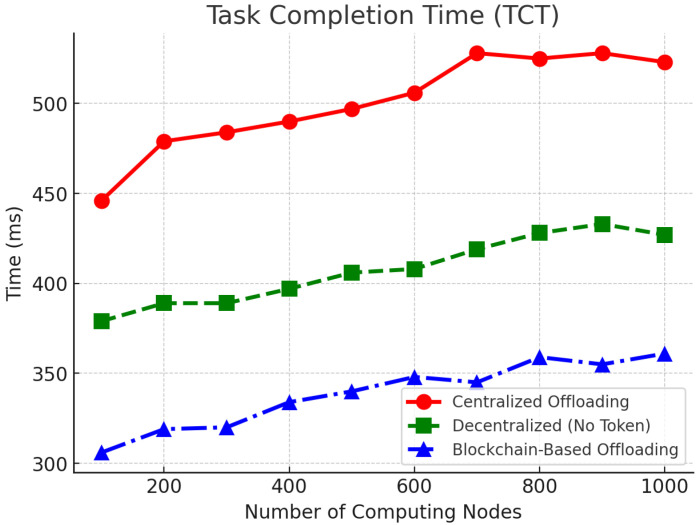
Task completion time (TCT) comparison across different computing nodes.

Figure 4 shows the task completion time (TCT) of the three schemes under different numbers of computing nodes. The blockchain-based offloading technique always shows the shortest task completion time, with latency reduced between 12.5% and 24.3% compared to centralized offloading. The decentralized mechanism without token incentives is also preferable to the centralized technique, but not as good as the blockchain-based mechanism. As the number of computing nodes increases, all methods experience a slight increase in task completion time due to communication overhead and congestion, but the blockchain method is the most stable.

**Figure 5:**
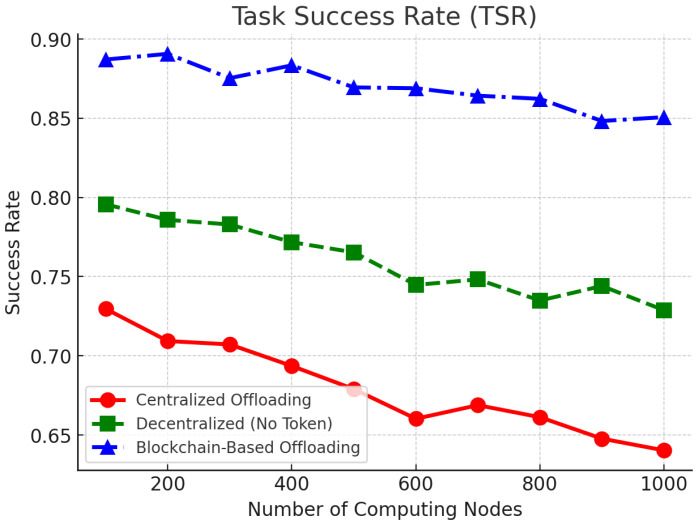
Task success rate (TSR) comparison across different computing nodes.

Figure 5 illustrates the task success rate (TSR) against the number of computing nodes. The blockchain-based solution achieves the highest success rate, 84.2–88.5%, 6.8–9.4% more than the decentralized model, and 15.7–23.1% more than the centralized model. The reason lies in the fact that adaptive resource allocation enables dynamic mapping of computational tasks to nodes with available resources. The centralized strategy is plagued by reduced success rates, mainly because of poor resource awareness and the potential for bottlenecks at congested nodes.

Our TSR under the stated settings is 84.2–88.5%. Direct numerical comparison is non-trivial because many vehicular/VEC studies primarily report end-to-end delay or throughput rather than an explicit task success rate (TSR), or they define “success/SLA satisfaction” under different deadlines and feasibility constraints. Representative works on mobility-/dependency-aware scheduling and VEC offloading (e.g., [9,10,21,22]) emphasize latency improvements with centralized or partially centralized coordination, while blockchain-oriented efforts (e.g., [13,14]) discuss accountability/incentives and system throughput but seldom provide a like-for-like TSR under dynamic vehicular settings. Within these caveats, our decentralized, incentive-compatible design attains TSR in the 84.2–88.5% range while explicitly accounting for on-/off-chain confirmation overheads and network volatility, indicating competitive reliability under comparable load and mobility regimes.

**Figure 6:**
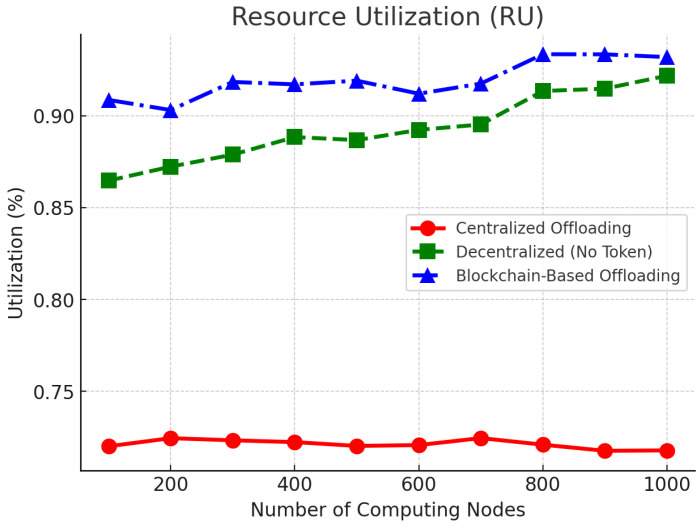
Resource utilization (RU) comparison across different computing nodes.

Figure 6 gives the resource usage (RU) of different computation nodes. The blockchain system optimizes computational efficiency to such an extent that the usage of resources is kept between 88.9% and 92.7%, far beyond that of the centralized system of around 71.8%. The economically incentivized–decentralized architecture slightly improves efficiency compared to the non-incentivized decentralized model, which results in resource utilization of 85.5–89.3%. This validates and reaffirms the fact that the token-based rewarding method in blockchain ensures a more efficient and fair resource supply approach.

**Figure 7:**
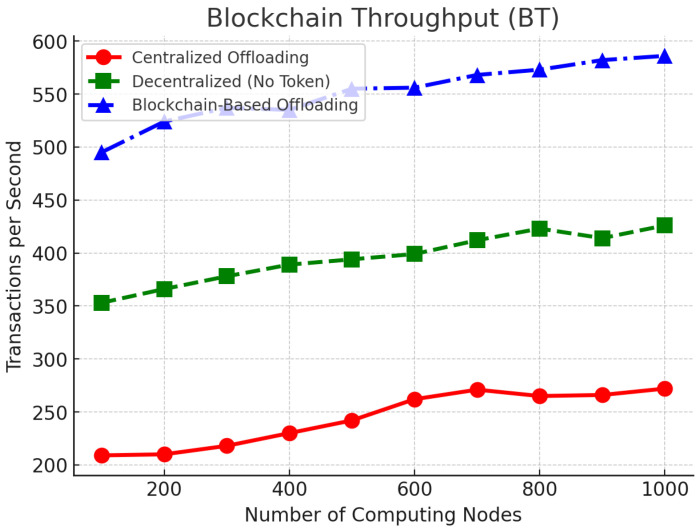
Blockchain throughput (BT) comparison across different computing nodes.

Figure 7 measures the blockchain throughput (BT) in terms of transactions per second processed. Our offloading protocol consistently achieves >480 transactions per second with a 10-second block interval, providing scalability for large-scale deployment. The decentralized approach without token incentives offers moderate throughput, peaking at 415 transactions per second, while the centralized model struggles to exceed 275 transactions per second, as it faces coordination bottlenecks. The results verify that the blockchain model is highly scalable and can efficiently allocate real-time computational tasks in an ITS environment.

To consolidate the main findings reported in Figure 4, Figure 5 and Figure 6 and the accompanying text, we collate the headline metrics for the three methods in Table 3. This summary mirrors our reporting conventions (mean values and ranges where appropriate) and keeps TCT in relative form when absolute baselines are not explicitly stated.

We summarize the main metrics using values reported in Section 6 (Figure 4, Figure 5 and Figure 6). As Table 3 indicates, the proposed approach (OURS) consistently reduces completion time relative to CEN, achieves TSR in the 84.2–88.5% range with clear gains over both baselines, and attains higher resource utilization while sustaining >480 TPS at $T_{\mathrm{block}}\;=\;10$ s. These trends align with the detailed plots in Figure 4, Figure 5 and Figure 6 and the statistical protocol in Section 6.1, and they are reflected again in the quantitative highlights summarized in Section 7.

The evaluation results confirm that the blockchain-based task offloading approach outperforms both centralized and non-tokenized decentralized approaches across all measured metrics. The decentralized nature of blockchain ensures dynamic resource allocation, reducing task completion time while maintaining a high task success rate. Token incentives further enhance computational resource utilization, ensuring sustainable participation from computing nodes. Additionally, the blockchain infrastructure enables high transaction throughput, making it suitable for large-scale intelligent transportation systems. These findings validate the feasibility of blockchain-driven decentralized task offloading as an efficient and scalable solution for ITS applications.

## 7. Final Remarks

In this paper, we addressed resource allocation and task offloading in decentralized, blockchain-based intelligent transportation systems (ITSs). We introduced a blockchain-driven paradigm with a token-based reward mechanism to incentivize fair and effective use of computational resources. Using a representative ITS setting, we showed that smart contracts enable task allocation in a transparent and secure manner. We built a simulation model and conducted extensive experiments to show that our scheme increases the task success ratio, reduces total completion time, and improves system resource utilization compared with centralized scheduling methods. We further validated the scalability of blockchain-based offloading, sustaining high transaction throughput and stable token distribution.

### 7.1. Quantitative Highlights

Our evaluation shows that the proposed approach reduces total completion time by 12.5–24.3%, achieves a task success rate of 84.2–88.5% (with consistent gains over the baselines), improves average resource utilization to 88.9–92.7%, and sustains >480 TPS at a block interval of $T_{\mathrm{block}}=10$ s. These gains hold across the tested network dynamics and workload intensities.

### 7.2. Limitations

Despite the above improvements, our study has several limitations: (i) a fixed block interval is assumed, whereas real deployments may require adaptive confirmation targets; (ii) the simulation-based network model cannot fully capture all field effects (e.g., interference, handovers, and RSU placement constraints); (iii) the staking and penalty parameters of PoS may be sensitive to market conditions and adversarial strategies; and (iv) confirmation delay and on-chain fees can affect tail latency under bursts or congestion.

### 7.3. Future Work

We plan to investigate (i) adaptive/variable block intervals and a batching/rollup-style settlement to reduce on-chain overhead; (ii) learning-based and *adaptive* offloading policies (e.g., DRL, decomposition-guided heuristics) for large-scale scenarios; and (iii) small-scale field trials with real RSU/V2V infrastructure to validate robustness and cost models in the wild.

## Figures and Tables

**Table 1:** Feature summary of representative related works. Y/P/N denote Yes/Partial/No; C = Conceptual, L = Limited.

Work (Domain)	Decent.	Net-Aware	Incent./Cons.	Overhd.
Cai et al. [9] (Vehicular)	N	Y	N	L
Liberati et al. [10] (Vehicular)	N	Y	N	L
Liu et al. [13] (BC, manuf.)	P	P	Y	P
Yu et al. [14] (Survey)	C	C	C	N
Tang et al. [21] (VEC)	P	Y	N	L
Mirza et al. [22] (IoT/Spark)	N	P	N	L
**This work (ITS)**	Y	Y	Y (PoS)	Y

**Table 2:** Notations.

Symbols	Descriptions
$V_{i}$	The $i^{th}$ vehicle in the scenario
*N*	Total number of vehicles in the system
*K*	Number of available computing nodes (including vehicles and RSUs)
$T_{j}$	The $j^{th}$ computational task
*M*	Number of tasks in the current scheduling window
$W_{j}$	Computational workload of task $T_{j}$ (GHz)
$S_{j}$	Data size of task $T_{j}$ (MB)
$D_{j}$	Task completion deadline (ms)
$P_{j}$	Payment offered for executing task $T_{j}$
$R_{j}$	Reward obtained for completing task $T_{j}$
$C_{k}$	Computational capacity of computing node *k*
$B_{k}$	Available bandwidth for node *k* (MB/s)
$P_{k}$	Packet loss probability of node *k*
$x_{j,k}$	Binary decision variable
$T_{j,k}$	Total execution time of task $T_{j}$ on node *k*
$S_{k}$	Task matching score for computing node *k*
α,β,γ,δ	Weight coefficients for task matching score
ϵ	Packet loss penalty factor
λ	Base reward rate
$B_{k}\left(t\right)$	Token balance of computing node *k* at time *t*
$R_{k}^{task}$	Reward for executing a task
$R_{k}^{block}$	PoS block reward
$P_{k}^{gas}$	Transaction cost for blockchain operations
$P_{penalty}$	Penalty for task execution failure
$T_{block}$	Blockchain block generation time
$N_{TX}$	Number of task transactions processed
$T_{throughput}$	Blockchain transaction throughput
$T_{confirm}$	Task confirmation time
$P_{finality}$	Probability of blockchain finality
$P_{k}^{block}$	A node being selected to confirm a transaction

**Table 3:** Key results from our study (text and Figure 4, Figure 5 and Figure 6). ↓ lower is better; ↑ higher is better.

Metric	OURS (Absolute)	vs DEC-NOINC	vs CEN
TCT ↓	–	–	−12.5% to −24.3%
TSR ↑ (%)	84.2–88.5	+6.8% to +9.4%	+15.7% to +23.1%
RU ↑ (%)	88.9–92.7	85.5–89.3 (DEC)	∼71.8 (CEN)
TPS ↑ (tx/s)	>480 @ $T_{\mathrm{block}}=10$ s	peak ≈415	<275

*Sources:* TSR range and relative gains from Section 6 text / Figure 4; RU from Figure 6 (88.9–92.7% for OURS; 85.5–89.3% for DEC-NOINC; ∼71.8% for CEN); TPS from Figure 7 (>480 tx/s for OURS, ∼415 tx/s peak for DEC-NOINC, <275 tx/s for CEN). Absolute TCT for baselines not stated; reported reductions are relative to CEN.

## Data Availability

The raw data supporting conclusions of this aticle will be made available by the authors on request.
